# Enhanced Hydrogen Generation Performance of Al-Rich Alloys by a Melting-Mechanical Crushing-Ball Milling Method

**DOI:** 10.3390/ma14247889

**Published:** 2021-12-20

**Authors:** Lixiang Zhu, Meishuai Zou, Xiaodong Zhang, Lichen Zhang, Xiaoxuan Wang, Tinglu Song, Shuo Wang, Xiaodong Li

**Affiliations:** 1 School of Materials Science and Engineering, Beijing Institute of Technology, Beijing 100081, China; 3220201235@bit.edu.cn (L.Z.); zoums@bit.edu.cn (M.Z.); 17854264182@163.com (X.Z.); 3120205586@bit.edu.cn (L.Z.); 18434360254@163.com (X.W.); song@bit.edu.cn (T.S.); bitlxd@bit.edu.cn (X.L.); 2 Experimental Center of Advanced Materials School of Materials Science & Engineering, Beijing Institute of Technology, Beijing 100081, China

**Keywords:** aluminum alloys, melting-crushing-ball milling, hydrogen generation performance, hydrogen storage

## Abstract

The main problem for the application of hydrogen generated via hydrolysis of metal alloys is the low hydrogen generation rate (HGR). In this paper, active Al alloys were prepared using a new coupled method-melting-mechanical crushing-mechanical ball milling method to enhance the HGR at room temperature. This method contains three steps, including the melting of Al, Ga, In, and Sn ingots with low melting alloy blocks and casting into plates, then crushing alloy plate into powders and ball milling with chloride salts such as NiCl_2_ and CoCl_2_ were added during the ball milling process. The microstructure and phase compositions of Al alloys and reaction products were investigated via X-ray diffraction and scanning electron microscopy with energy dispersed X-ray spectroscopy. The low-melting-point Ga-In -Sn (GIS) phases contain a large amount of Al can act as a transmission medium for Al, which improves the diffusion of Al to Al/H_2_O reaction sites. Finer GIS phases after ball milling can further enhance the diffusion of Al and thus enhance the activity of Al alloy. The hydrogen generation performance through hydrolysis of water with Al at room temperature was investigated. The results show that the H_2_ generation performance of the Al-low-melting point alloy composite powder is significantly higher than the results reported to date. The highest H_2_ generation rate and H_2_ conversion efficiency can reach 5337 mL·min^−1^·g^−1^ for the hydrolysis of water with 1 g active alloy.

## 1. Introduction

Nowadays, economic growth remains desperately dependent on fossil fuels. However, the non-renewability and environmental problems of fossil fuels led to the urgent demand for clean and renewable energy sources [1,2,3,4]. H_2_ has gradually taken as future energy due to its high energy density, non-polluting combustion products, and abundance in nature [5,6,7,8]. There are currently two major problems for hydrogen energy applications: one is the H_2_ preparation methods. In the past, many methods such as fossil fuel reforming [9,10,11], water electrolysis [12,13], photocatalysis [14,15], and biological H_2_ generation [16,17] have been developed to produce H_2_, but these methods have some disadvantages such as low conversion efficiency, high costs, and environmental pollution. The second problem is H_2_ transportation with difficult storage and transportation costs [18,19]. Therefore, it is necessary to find new methods for H_2_ generation.

**Figure 1:**
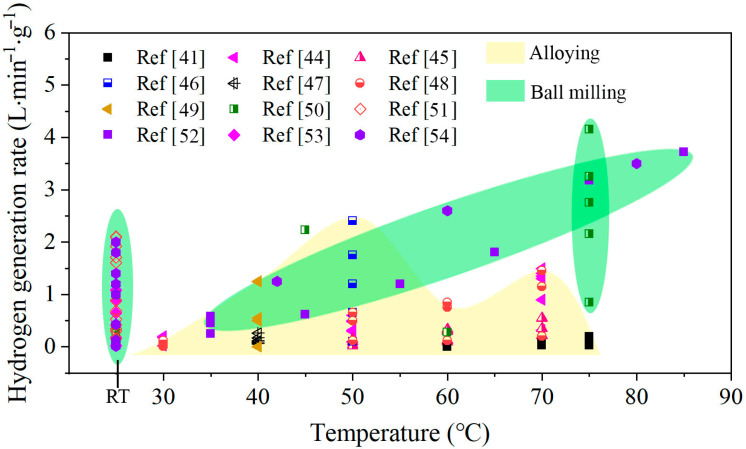
Hydrogen generation rate of various active Al composites reacted with water at different temperatures [41,44,45,46,47,48,49,50,51,52,53,54].

Hydrogen can also be indirectly stored and transported by reactive metals, such as Zn [20,21], Mg [22,23], and Al [24,25,26], and then released via hydrolysis reaction of water. In this way, the real-time and on-demand production and use of hydrogen energy can be realized [27]. Aluminum has received much attention for H_2_ generation due to its high energy density, light weight, and abundance in nature [28]. Moreover, Al can be fully recycled from the byproduct of the hydrogen evolution reaction by the electrolysis process [29]. However, pure Al cannot react with water at room temperature because the dense oxide film formed on the Al surface inhibits the Al/H_2_O reaction. To remove the surface oxide film and promote Al/H_2_O reactions, many methods have been adopted to activate Al. One is dissolving Al by acidic or alkaline solution [30,31,32,33], but acidic or alkaline solutions are corrosive and can cause apparatus corrosion. Another method is alloying Al powders with metal additives by mechanical ball milling [34,35,36,37,38], but this method has a long mechanical ball milling time and high cost. Another way is alloying Al with other metals by melting [39,40,41,42,43]. The realization of real-time and on-demand production of H_2_ mainly depends on the HGR of Al/H_2_O reaction, which should meet the requirement of quick response and high H_2_ production rate supply. However, the Al alloy prepared with these methods has slow reaction rates and low H_2_ generation yield at room temperature, as shown in Figure 1. The Al using ball milling method shows higher activities than the alloying, especially at room temperature. However, the high hydrogen rate achieved is at the expense of hydrogen production using more additives. Therefore, it is important to find new ways to activate Al to produce H_2_ with a high generation rate at room temperature.

Previous results found that alloying Al with low-melting-point metals (Ga, In, Sn, Bi) to form active Al-based alloy can achieve a high H_2_ generation performance [28,43,46,48,55,56]. The activity of Al in the alloy can be attributed to micro-galvanic cells [48,57] or diffusion of Al through the low-melting-point liquid phase [39]. In an Al alloy and water system, Al and other metal will form a micro-galvanic cell where Al takes as the anode, and other metals take as the cathode. The corrosion rate of Al depends strongly on the electrode potential of Al and its contacting metals [48]. Low-melting-point phases enabled the observed reaction upon liquefaction by providing a means of transport for Al in the alloys to reach reaction sites [39,58]. Wang et al. [59] prepared Al-Ga-In-Sn alloys by melting Al with low-melting-point metals (Ga, In, Sn) and found that the low-melting-point metals can greatly improve the H_2_ generation performance of Al alloys. Of these, Al-1.75Ga-0.75In-0.5Sn has superior H_2_ generation performance. The H_2_ conversion efficiency is close to 100%, along with the highest H_2_ generation rate up to 280 mL·min^−1^·g^−1^ Al at 40°. Wang et al. [53] prepared some Al alloys with different low-melting-point metal (Ga, In, Sn) compositions by mechanical ball milling. They found that the low-melting-point compositions and structure of the Al alloys have a great influence on the H_2_ generation performance. The activity of quaternary Al-Ga-In-Sn alloy is greatly improved compared with the ternary-activated Al system due to the presence of low-melting-point phases as the lower melting point [44,53]. Of these, the H_2_ conversion efficiency and highest H_2_ generation rate of Al-3Ga-3In-5Sn alloys can reach 99.23% and 1081 mL·min^−1^·g^−1^. In addition, some researchers found that adding some salt additives can also improve the H_2_ generation performance of Al [60,61]. Fan et al. [62] prepared a series of Al-10Li-5NiCl_2_/NaBH_4_ mixtures by mechanical ball milling. The H_2_ generation rate and H_2_ conversion efficiency of the Al mixture gradually decrease as the content of NiCl_2_ increases, so NiCl_2_ greatly influences the H_2_ generation performance of the Al mixture. The H_2_ conversion efficiency and the highest H_2_ generation rate of Al-10Li-5NiCl_2_ mixture can reach 97% and 379 mL·min^−1^·g^−1^ Al.

However, the activation of Al is currently limited to melting and ball milling individually; it is difficult to achieve a further breakthrough for the H_2_ generation performance of Al alloys. Therefore, how to improve the H_2_ generation rate and H_2_ conversion efficiency of Al alloys has always been a challenging task. In this study, we proposed a coupling method with melting, mechanical crushing, and ball milling (MMB). The melting step is to make an alloy, the mechanical crushing step is to make alloy powder, and the ball milling step is to active Al alloy. Two Al alloy systems (Al-3Ga-3In-3Sn [53] and Al-3.8Ga-1.5In-0.7Sn alloys [40,45]) were selected for their poor mechanical properties (can be broken easily by hand), suitable hydrogen generation properties at room temperature (but with low HGR), and high Al content (>90%). NiCl_2_ and CoCl_2_ were chosen as the catalysts for their suitable performance in activating Al [62,63] and Mg [23,64]. The H_2_ generation performance of active Al alloys using different methods was investigated. The structure of the Al alloys and their hydrolysis reaction products were also characterized.

## 2. Experimental Details

### 2.1. Preparation of Al Alloys

Al ingots (Aladdin, Shanghai, China, 99.99% purity), Ga blocks (Aladdin, Shanghai, China, 99.9% purity), In blocks (Aladdin, Shanghai, China, 99.9% purity), Sn particles (Aladdin, Shanghai, China, 99.9% purity), NiCl_2_ powder (Aladdin, Shanghai, China, 99.8% purity), and CoCl_2_ powder (Aladdin, Shanghai, China, 99.9% purity) were used as starting materials. The first step is melting. Al-Ga-In-Sn alloys were prepared by melting the metals at a calculated mass ratio in a graphite crucible in a resistance furnace at 900 °C with isothermal heating for 1 h. The melting process was conducted under an argon atmosphere. The cast melt was then divided into pieces and allowed to cool at room temperature. The second step is mechanical crushing. The Al alloys pieces prepared were crushed into powders using a mechanical crusher (Xulangmachinery, Guangzhou, China, XL-10B), and a 300-mesh sieve was used to screen the powders. The third step is ball milling, the ball milling parameters was listed in Table 1. Mechanical crushing powder and salts were mixed in a ball mill jar. The ball milling experiment was conducted under an argon atmosphere with slightly positive pressure using stainless steel ball. Th ball-to-powder ratio was set as 40:1, and the ball milling experiment was conducted for 1 h under 500 rpm. All of the prepared powders were stored in n-hexane.

### 2.2. Hydrogen Generation Performances of Al Alloys

**Figure 2:**
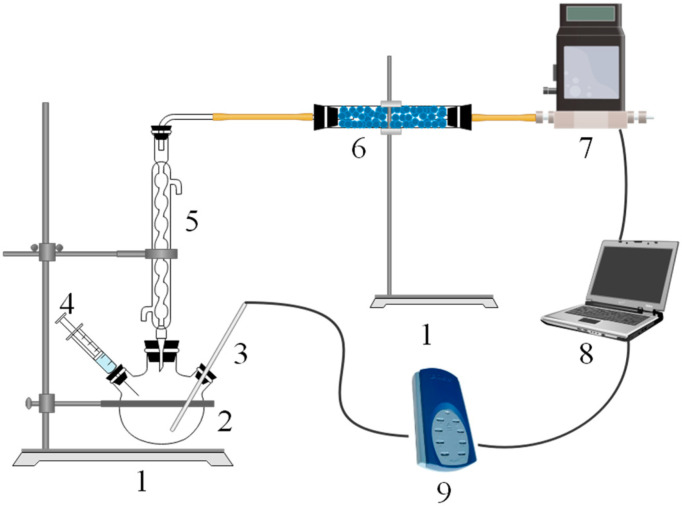
Schematic diagram of the experimental setup. 1—iron frame platform, 2—three-neck flask, 3—thermometer, 4—syringe, 5—condenser tube, 6—drying tube, 7—gas mass flowmeter, 8—laptop, and 9—thermocouple data logger.

The setup used in the H_2_ generation experiments is shown in Figure 2. A 500 mL three-neck flask was placed on the iron frame platform using a tube clamp. A total of 1.0 g of active Al alloy or alloy composites were placed in the reactor, and the neck was sealed. Next, we injected 20 mL water into the reactor with 2 s without mixing or heating during the Al/H_2_O reaction. The resulting hydrogen was cooled and dried after passing through the condenser and drying tubes. The H_2_ generation yield and rate were detected by a gas mass flowmeter. All the hydrogen generation experiments were conducted at the same setup and reaction conditions. So, we did not take other factors such as the rate of H_2_O injection, the volume of apparatus, mixing rate of water and alloy, etc., into account. For each experiment, the temperature change of the Al/H_2_O reaction can be detected by a thermocouple inserted into the reaction area and recorded using a TC-08 temperature recorder. All of the temperature data and hydrogen generation data were recorded simultaneously. The reaction products were collected and vacuum dried before being analyzed.

### 2.3. Microstructure Analysis of Al Alloys

To study the phase compositions, Al alloys, together with the reaction products, were analyzed by X-ray diffraction (XRD) using a Rigaku D/max 2400 diffractometer (PANalytical, Almelo, Netherlands) with monochromated CuKα radiation. The scans were collected over a 2θ range of 10°–120°. The microstructure of Al alloys together with the reaction products was characterized using a scanning electron microscope (SEM, Hitachi, Tokyo, Japan) with a Quanta 600 EDX (Energy Dispersed X-ray) system. All the samples were coated with Au before analysis to avoid the possible poor conductivity as the oxidation on the sample surface. The back-scattered mode was adopted for the observation of precipitated phase of low-melting-point metal. To analyze the chemical states of Al and O of the Al alloys, X-ray photoelectron spectroscopy (XPS, ULVAC-PHI Inc., Chigasaki, Japan) was operated at 15 kV with an alumina target (Al-Kα, h*v* = 1486.6 eV). To analyze the Ni and Co in the samples with low content, the Ni and Co K-edge XAFS measurements were performed at the beamline 4B9A of the Beijing Synchrotron Radiation Facility (BSRF) at the Institute of High Energy Physics (IHEP), Chinese Academy of Sciences (CAS).

## 3. Results and Discussion

### 3.1. Characterization of Al Alloys

#### 3.1.1. XRD Analysis

**Figure 3:**
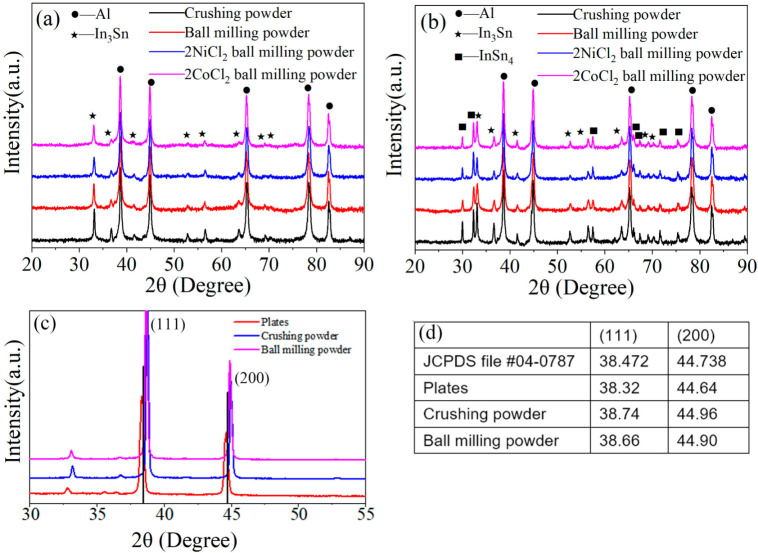
The XRD patterns of Al alloys: (**a**) Al3.8Ga-1.5In-0.7Sn and (**b**) Al-3Ga-3In-3Sn. (**c**) XRD pattern of Al-3.8Ga-1.5In-0.7Sn alloy under different conditions between 30° and 55°, (**d**) the 2θ data of (111) and (200) of Al-3.8Ga-1.5In-0.7Sn alloy.

Figure 3 shows the XRD patterns of Al alloys with different compositions. Characteristic signature sequences of Al phase (JCPDS file #04-0787) and In_3_Sn (*β*, JCPDS file #07-0345) intermetallic compound are observed for Al-3.8Ga-1.5In-0.7Sn alloy. However, intermetallic compounds In_3_Sn and InSn_4_ (*γ*, JCPDS file #48-1547) are both found at the same time besides Al (Ga) solid solution for Al-3Ga-3In-3Sn alloy as the increase in In/Sn ratio [44]. The phases of both alloys are not changed after ball milling, but the peak intensity of the In-Sn intermetallic compound decrease after ball milling, especially for Al-3.8Ga-1.5In-0.7Sn alloy (Figure 3a). Characteristic peaks of NiCl_2_ and CoCl_2_ were not detected, which is most likely due to the low content or overlapping by other peaks.

The XRD pattern of Al-3.8Ga-1.5In-0.7Sn alloy under different conditions between 30° and 55° and the data are shown in Figure 3c,d. Al peaks in (111) and (200) of alloy plate move left a little, which suggests that Ga atoms successfully entered into Al crystal lattice to form Al(Ga) solid solution (crystal) [42,44,46]. We take (111), for example, the interplanar distance in (111) of Al grain has increased by about 23.3% with the addition of metal Ga. However, the peaks of Al alloy move to the right after crushing and ball milling. This phenomenon indicates that crushing and ball milling processes have a great effect on crystal structures.

#### 3.1.2. Morphology Characterization

**Figure 4:**
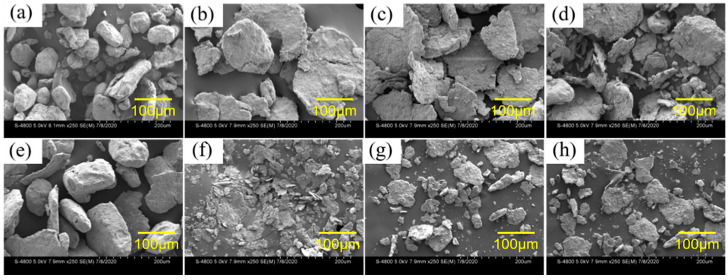
SEM images of the two kinds of active Al alloy powders: (**a**–**d**) Al-3.8Ga-1.5In-0.7Sn and (**e**–**h**) Al-3Ga-3In-3Sn. (**a**,**e**) crushing powder, (**b**,**f**) ball milling powder, (**e**,**g**) ball milling powder with 2% NiCl_2_, (**d**,**h**) ball milling powder with 2% CoCl_2_.

**Figure 5:**
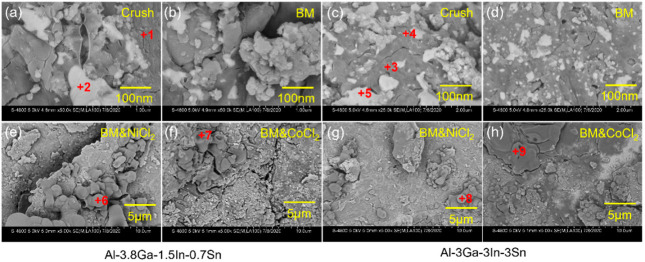
The amplified SEM images of Figure 4: (**a**,**b**,**e**,**f**) Al-3.8Ga-1.5In-0.7Sn, (**c**,**d**,**g**,**h**) Al-3Ga-3In-3Sn.

Figure 4 and Figure 5 show the SEM images of Al alloys with different compositions. The mechanical crushing powder is rod-shaped with a diameter of 20–150 μm for both alloy systems. The shape changes from spherical or rod-shaped (Figure 4a,e) to flake-shape after ball milling (Figure 4b–d,f–h). Ball milling can only change the morphology of Al-3.8Ga-1.5In-0.7Sn alloy but cannot refine the powder, as shown in Figure 4a and Figure 3b. The addition of chloride leads to only a slight size refinement effect (Figure 4b–d). However, the Al-3Ga-3In-3Sn powders were greatly refined after ball milling, as shown in Figure 4e,f. The size of the Al-3Ga-3In-3Sn powders changes from ~150 μm for crushing powder to 10–40 μm after ball milling (Figure 4e,f). After adding chloride, the powder gathers together during ball milling, and the powder size increases, as shown in Figure 4g,h.

The high magnification images of active Al alloy powder are shown in Figure 5 in correspondence with Figure 4. The low-melting-point Ga-In-Sn (GIS) phases can be seen on the surface of crushing powders for both alloys seen in Figure 5a and Figure 4c. For the Al-3.8Ga-1.5In-0.7Sn crushing powder, as shown in Figure 5a, low-melting-point alloy phases are observed as large as no more than 1 μm. In addition, the oxide film on the surface is dehiscent and curly at the edge, and this may be the effect of low-melting-point alloy, which causes discontinuity of the oxide film on the surface of Al grains [65]. After ball milling for 1 h, the low-melting-point alloy phases are refined with sizes of 100–200 nm. There are lots of finer low-melting-point alloy phases on the surface of Al-3Ga-3In-3Sn crushing powder, as Figure 5c shows. The dehiscent and curly edges are also observed near low-melting-point alloy phases. After ball milling for 1 h, the low-melting-point metal phases on the powder surface become finer and more dispersed, as Figure 5d shows with a size of ~ 20 nm. The composition of Al-based area and low-melting-point metal phases shown in Table 2 revealed that the Al-based phase mainly contains Al and Ga, besides a small amount of In and Sn. This might be caused by the quenching of In and Sn into Al during casting [46]. In addition, low-melting-point phases containing a large amount of Al can improve the diffusion of Al to Al/H_2_O reaction sites and result in a continuous reaction of Al and H_2_O [39,58,66]. Thus, finer GIS phases can further enhance the activity of Al. However, the atom ratio of In and Sn is not 3/1 or 1/4, which may be caused by oxidation.

The surfaces of the Al alloy are smooth after ball milling but have some big cracks for these two alloys, as shown in Figure 5b,d. More defects and cracks are found after ball milling on the addition of NiCl_2_ and CoCl_2_ for these two alloys. However, no obvious Ga-In-Sn phases were seen on the powder surface, maybe with finer size, but with some new phase generated instead, as shown in Figure 5e–h. The composition listed in Table 2 shows that the new generated phase contains high content of O, with an Al/O atom ratio higher than 1.5 and with a high Cl content at the same time.

#### 3.1.3. H_2_ Generation Performance of Al Alloys

**Figure 6:**
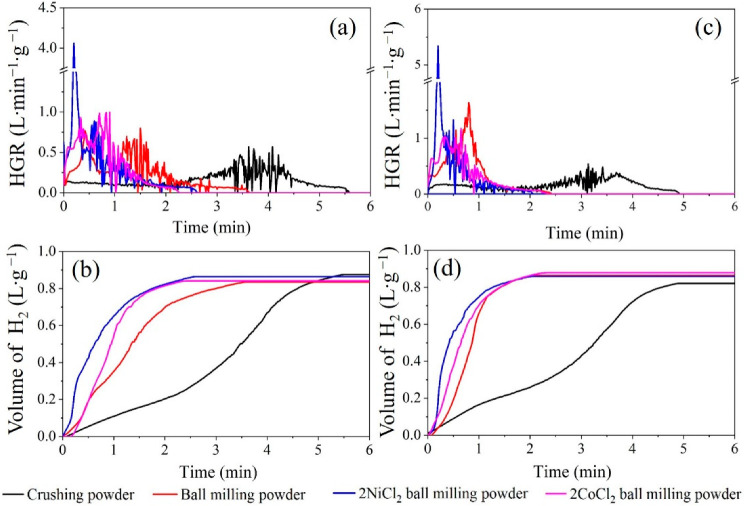
The H_2_ generation curves of Al alloys: (**a**,**b**) Al-3.8Ga-1.5In-0.7Sn and (**c**,**d**) Al-3Ga-3In-3Sn.

The H_2_ generation performances of the two selected Al alloys with tap water were tested at room temperature. Figure 6 shows the H_2_ generation yield and H_2_ generation rate of two Al alloys. It should be noticed that all the data are collected after taking the theoretical aluminum content into consideration. The activity of the Al alloy powder is greatly improved after ball milling for both alloys. The addition of NiCl_2_ and CoCl_2_ during ball milling can further enhance the generation performance of the Al alloy composite powders. However, the activities of these two alloys under different technological processes are different.

The activity of Al-3.8Ga-1.5In-0.7Sn can be gradually promoted by ball milling and ball milling with NiCl_2_ and CoCl_2,_ as revealed by the shortened reaction duration (Figure 6a). The H_2_ generation rate varies markedly under different activation methods. Ball milling is effective in shortening the reaction time as the grain refinement of the GIS phase, as shown in Figure 5a–d. The highest HGR (HGR) occurs in advance and with a higher value versus the crushing powder. The HGR reaches over 4000 mL·min^−1^ for both ball milling alloy powders with NiCl_2_ as shown in Figure 6a,c and Table 3. The reaction durations are greatly shortened after ball milling with NiCl_2_ and CoCl_2_. This is rarely achieved for most active Al alloys at room temperature [41,44,45,46,47,48,49,50,51,52,53,54]. The hydrogen yields of active Al-3.8Ga-1.5In-0.7Sn alloy powders are similar under different activation methods, although with different HGR and reaction durations, as shown in Figure 6b. The highest H_2_ generation volume occurs for alloy powder after ball milling with NiCl_2_.

The activity of Al-3Ga-3In-3Sn alloy and Al-3Ga-3In-3Sn alloy powders greatly improved after ball milling. The addition of NiCl_2_ and CoCl_2_ during ball milling can only increase the maximum HGR and H_2_ generation volume, but the overall reaction duration cannot be shortened (Figure 6b,c). Both the highest HGRs for these two alloys were achieved after ball milling with NiCl_2_. The highest HGR can reach 4060 mL·min^−1^ and 5337 mL·min^−1^ for Al-3.8Ga-1.5In-0.7Sn-2NiCl_2_ and Al-3Ga-3In-3Sn-2NiCl_2_ ball milling powders, respectively, which is rarely reported. Ball milling with chloride can dramatically improve the HGR and with similar hydrogen generation volume as the data listed in Table 3. In addition, the hydrolysis reaction duration is greatly shortened from about 5 min to about2 min at room temperature for ball-milled Al-3Ga-3In-3Sn alloy powders. This high-HGR property of the alloy composites shows a promising application in the on-demand production and use of hydrogen energy.

In this work, the conversion yields of alloys and alloy composites should take the Al content and purity into consideration. Here, we first calculate the theoretical hydrogen generation volume for the alloys and alloy composites. Then the conversion yield is obtained by dividing the experimental value by the theoretical one. The calculated data in Table 3 show that the Al-3Ga-3In-3Sn alloy powders after ball milling with 2% CoCl_2_ for 1 h show a higher H_2_ conversion yield, which can reach 82.68%. However, the highest conversion yield for Al-3.8Ga-1.5In-0.7Sn alloys occurred after ball milling with 2% NiCl_2_ (75.77%). The conversion yields are all lower than 85%, which may be the result of the partial oxidation of Al in the samples as detected in the SEM and EDX.

#### 3.1.4. XPS Analysis

**Figure 7:**
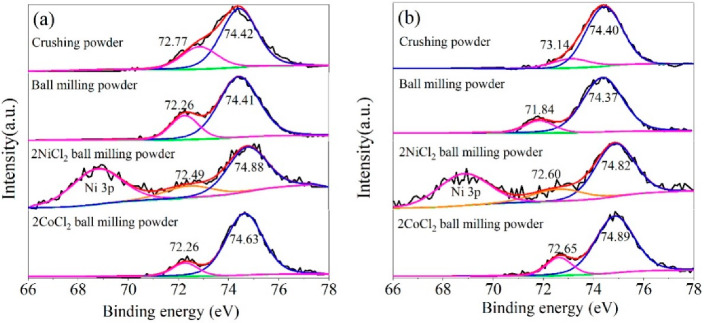
Curve-resolved XPS of the Al 2p region for the Al alloy powder: (**a**) Al-3.8Ga-1.5In-0.7Sn and (**b**) Al-3Ga-3In-3Sn.

Figure 7 shows the Al 2p spectra of Al-3.8Ga-1.5In-0.7Sn and Al-3Ga-3In-3Sn active alloys. The results reveal the presence of Al and Al^3+^ for all the active Al alloys, which is consistent with the results analyzed by EDX. XPS has limited detection depth, and a large amount of Al_2_O_3_ on the surface is detected, although only a small amount of Al_2_O_3_ exists in the activated alloy powders. The SEM images in Figure 5 show that oxidation is generated on the Al alloy surface, and the active Al is exposed as the non-smooth surface (with cracks).

Figure 7 shows that both the binding energies of Al_2_O_3_ 2p and Al 2p shifted versus the standard value (74.7 eV for Al_2_O_3_ 2p and 72.65 eV for Al 2p), which is related to changes in the chemical environment of Al_2_O_3_ and Al. For two different Al alloys, the binding energy of Al 2p for crushed powder is significantly higher than other powders, and the hydrogen generation activity of crushed powder is significantly lower than other powders. The higher the binding energy of Al 2p implies that it is harder to lose electrons and higher chemical stability; thus, the hydrogen generation activity of crushing powder is lower. The binding energy of the Al 2p is relatively low for ball-milled Al alloys and alloy composites. The binding energy of Al 2p for ball milling powder is the lowest for both alloys (as seen in Figure 7) but with a lower hydrogen generation rate. This means the added NiCl_2_ and CoCl_2_ may have other activation mechanisms for ball milling Al alloy composites.

#### 3.1.5. EXAFS Analysis

**Figure 8:**
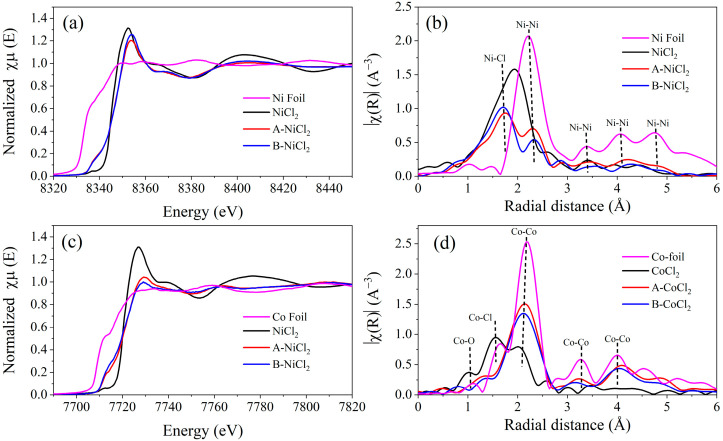
Normalized XANES spectra of Ni and Co K-edge and (**a**,**c**) and Fourier-transformed extended X-ray absorption fine structure (EXAFS) (**b**,**d**) of the ball milling alloy with salts. A is Al-3.8Ga-1.5In-0.7Sn, B is Al-3Ga-3In-3Sn.

EXAFS was conducted to characterize Ni and Co for their low content in the samples. Figure 8a,b shows normalized Ni K-edge X-ray absorption near-edge structure (XANES) spectra of the Al alloy-chloride composites as well as the reference samples of Ni foil and NiCl_2_. The absorption edge (E_0_) for pure Ni laying at 8333 eV. The Ni shows an absorption edge shifting toward lower energy compared with NiCl_2_, which means the partial reduction in NiCl_2_ by Al alloy occurred during ball milling with an intermediate state between metallic Ni and NiCl_2_ [67,68]. Fourier transformation of the XAFS spectra showed that the ball-milled samples have two domain peaks at around 1.56 Å and 2.30 Å (Figure 8b), which are recognized as Ni-Cl and Ni-Ni distances [69]. The above phenomenon reveals that Ni is generated during the ball milling process. A similar phenomenon occurred for the ball milling Al alloy with CoCl_2,_ as shown in Figure 8c,d. The results qualitatively indicate the strong influence of ball milling with Al alloy on the variation in the chemical bonding of the Ni and Co atom and confirm the exiting of metallic Ni and Co after ball milling. The existence of Ni and Co in the hydrolysis reaction system can act as a cathode, and thus the corrosion of Al in the alloy powder composites can be accelerated with a higher HGR, as shown in Figure 6 and Table 3. The different electrode potentials for Ni and Co may be the reason for the different hydrogen generation performance, as shown in Figure 6.

### 3.2. Characterization of the Hydrolysis Products of Al Alloys

#### 3.2.1. XRD Analysis

**Figure 9:**
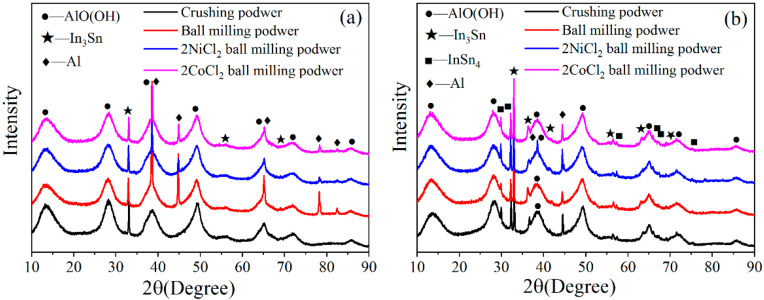
The XRD patterns of the hydrolysis products of Al alloys: (**a**) Al-3.8Ga-1.5In-0.7Sn and (**b**) Al-3Ga-3In-3Sn.

Figure 9 shows the XRD patterns of the hydrolysis products in the reaction of Al alloy powder with water. The hydrolysis products of the two alloy powders mainly consist of AlO(OH) [46,53], In and Sn intermetallic compounds, and a small amount of unreacted Al (except for the crushing powder of Al-3.8Ga-1.5In-0.7Sn). In and Sn can form In_3_Sn and InSn_4_ intermetallic compounds for Al-3Ga-3In-3Sn while In and Sn only form In_3_Sn intermetallic compounds for Al-3.8Ga-1.5In-0.7Sn. This result is consistent with the XRD pattern of the Al alloy. In-Sn intermetallic compounds are not changed during the hydrolysis reaction, which means that they are not involved in the Al/H_2_O reaction but rather simply act as catalysts. The unreacted Al peaks are detected for alloys, and this is consistent with the conversion yield data of Al-3.8Ga-1.5In-0.7Sn alloy powders in Table 3.

#### 3.2.2. SEM Observation

**Figure 10:**
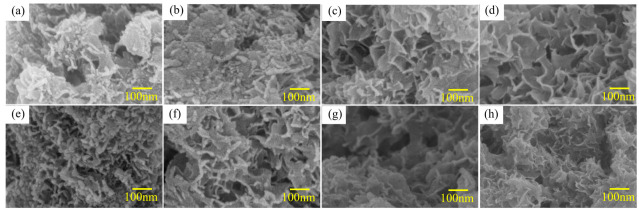
The SEM images of the hydrolysis products of Al alloys: (**a**–**d**) Al-3.8Ga-1.5In-0.7Sn ball active powder and (**e**–**h**) Al-3Ga-3In-3Sn active powder.

Figure 10 shows the SEM images of the hydrolysis products in the reaction of Al alloy powder with water. The products show different morphologies for different alloys and alloy composites active in different activation methods. The products’ morphologies for Al-3.8Ga-1.5In-0.7Sn alloy and composites are shown in Figure 10a–d. Dense products generated with spheroid and wormlike structures on the surface are observed for products for crushing powder and ball milling powder in Figure 10a,b. However, the products’ morphologies change into porous structures with holes after ball milling with chloride salts, as can be seen from Figure 10c,d. Dense products generated with spheroid and wormlike structures can only be observed for Al-3Ga-3In-3Sn crushing powder (see in Figure 10e). The products of the rest of active alloy and alloy composites are all porous structures with holes, as seen in Figure 10f–h.

The boehmite particles and hydrogen gas are produced at the same time. If the reaction is not rapid enough, a layer of boehmite will cover aluminum particles, and as the layer thickens, the reaction would decline [35]. As the hydrogen is generated in between boehmite and aluminum and then flows outwards, boehmite particles are repulsed, and the boehmite layer fails to surround aluminum particles [35]. Higher HGR thus can lead a strong powder to remove the newly generated boehmite and promote the exposure of fresh Al, and consequently with a higher HGR and products with porous structure.

## 4. Conclusions

In this study, a coupling method using melting, mechanical crushing, and ball milling (MMB) was used to prepare high active Al alloys and Al alloy composite powders. The hydrogen generation performance of Al alloy with water was investigated at room temperature. The microstructure and phase compositions of Al alloys and reaction products were investigated via XRD, SEM, XPS, and EXAFS analyses. The results are summarized as follows:

1. The H_2_ generation performance of the alloy prepared by the MMB method is significantly higher than the individual melting or ball milling method. The highest HGRs can reach 5337 mL·min^−1^·g^−1^ and 4060 mL·min^−1^·g^−1^ for Al-3.8Ga-1.7In-0.5Sn and Al-3Ga-3In-3Sn alloy powder, respectively.
2. The Ga-In-Sn phase plays an important role in promoting the Al/H_2_O reaction. The GIS phases contain a large amount of Al to improve the diffusion of Al to Al/H_2_O reaction sites. The grain refinementation after ball milling can further enhance the activity of Al and resulting in a higher HGR and continuous reaction of Al and H_2_O.
3. The lower binding energy of Al 2p after ball milling reveals the high reactivity of Al. The existence of metallic Ni and Co after ball milling Al alloy powder with NiCl_2_ and CoCl_2_ may act as a cathode and accelerate the corrosion of Al in the alloy powder composites. In this circumstance, the hydrolysis of water with Al can reach a higher HGR.

This work mainly focuses on the new coupled preparing method and the hydrogen generation rate. The MMB method is an economical method that avoids the expensive atomization powder process. Using this method, a high hydrogen generation rate of Al/H_2_O reaction can be achieved for the future application of real-time and on-demand production of H_2_.

## Figures and Tables

**Table 1:** Ball milling parameters.

No.	Alloy	Alloy Weight (g)	Ball Milling Time	rpm	NiCl_2_ (g)	CoCl_2_ (g)
1	Al-3.8Ga-1.5In-0.7Sn	20	0 h	500	0	0
2		20	1 h	500	0	0
3		20	1 h	500	4	0
4		20	1 h	500	0	4
5	Al-3Ga-3In-3Sn	20	0 h	500	0	0
6		20	1 h	500	0	0
7		20	1 h	500	4	0
8		20	1 h	500	0	4

**Table 2:** Compositions of Al alloys obtained using EDX.

Spectrum	Elements (at.%)							
	Al	Ga	In	Sn	Ni	Co	Cl	O
1	63.42	2.85	2.85	0.00	0.00	0.00	0.00	30.89
2	79.82	6.13	1.83	0.63	0.00	0.00	0.00	11.59
3	77.76	5.72	0.00	0.00	0.00	0.00	0.00	16.52
4	49.63	1.72	1.95	1.50	0.00	0.00	0.00	20.98
5	49.63	0.95	2.06	1.32	0.00	0.00	0.00	46.04
6	25.43	0.31	0.07	0.06	0.20	0.00	4.81	69.12
7	27.92	0.00	0.32	0.24	0.00	0.16	2.38	68.98
8	43.06	0.00	0.13	0.08	0.04	0.00	3.18	53.52
9	34.40	0.61	0.29	0.22	0.00	0.12	1.68	62.68

**Table 3:** The H_2_ generation performance of Al alloys.

Alloy	Alloy Powder	Volume of Hydrogen (mL∙g^−1^)	Conversion Yield (%)	Highest HGR (mL·min^−1^∙g^−1^)	Reaction Time (min)	Average HGR (mL·min^−1^∙g^−1^)
Al-3.8Ga-1.5 In-0.7Sn	Crushing powder	858	73.35	569	5.60	153.21
	BM powder	857	73.26	798	3.63	236.09
	BM-NiCl_2_ powder	869	75.77	4060	2.63	330.42
	BM-CoCl_2_ powder	828	72.20	995	2.27	364.76
Al-3Ga-3In-3Sn	Crushing powder	863	70.20	539	4.93	93.31
	BM powder	812	71.70	1171	2.35	345.53
	BM-NiCl_2_ powder	864	77.82	5337	2.07	417.39
	BM-CoCl_2_ powder	918	82.68	1634	2.43	377.78

## Data Availability

Data sharing not applicable.
